# Non-Malignant T Cells as Determinants of Immunotherapeutic Response in Chronic Lymphocytic Leukemia: Towards Personalized Strategies

**DOI:** 10.32604/or.2026.081365

**Published:** 2026-09-14

**Authors:** Agata Kosmaczewska, Lidia Ciszak

**Affiliations:** Department of Experimental Therapy, Hirszfeld Institute of Immunology and Experimental Therapy, Polish Academy of Sciences, Wroclaw, Poland

**Keywords:** Chronic lymphocytic leukemia (CLL), T cells, anti-tumor immunity, immunotherapy, T cell-based therapies

## Abstract

Chronic lymphocytic leukemia (CLL) is a biologically heterogeneous B cell malignancy in which non-malignant T lymphocytes constitute a critical component of the tumor microenvironment and significantly influence disease evolution and the therapeutic response. Growing evidence suggests that CLL-associated T cells not only participate in the antitumor response but also activate signals that promote the development of CLL subclones. Although novel targeted therapies, such as Bruton’s tyrosine kinase (BTK) inhibitors, BTK degraders, B-cell lymphoma 2 (BCL-2) inhibitors, T cell engagers, immune checkpoint inhibitors, and adoptive T cell therapy have different mechanisms of action, they affect the T cell compartment in addition to targeting CLL cells. Therefore, in-depth knowledge of the role of T cells in the development and progression of CLL is essential for proper stratification of the benefit-to-risk ratio with regard to immunotherapeutic strategies. This review comprehensively summarizes current knowledge on alterations within the T cell compartment in CLL and discusses the clinical implications, regarding clinical course and response to immunotherapy.

## 1 Introduction

According to the Surveillance, Epidemiology, and End Results Program, chronic lymphocytic leukemia (CLL) is the most common type of leukemia in adult patients (with a mean age at diagnosis of 70 years), accounting for 4.7 cases per 100,000 inhabitants per year [1]. CLL is characterized by the malignant proliferation of mature monoclonal CD5^+^ B lymphocytes circulating between lymph nodes (LNs) and the peripheral blood (PB) and is associated with failed apoptosis and increased proliferation. CLL cell growth is supported by surrounding cells in the LNs, including stromal cells, myeloid-derived suppressor cells (MDSCs), and supportive T cell subsets such as follicular helper T (Tfh) cells and regulatory T cells (Tregs), which together create the tumor microenvironment (TME). The immunosuppressive components of the TME include CD4 and CD8 T cells displaying impaired antitumor properties, thereby allowing CLL cell growth [2,3]. The clinical course of CLL is characterized by heterogeneity, ranging from asymptomatic/stable disease to a symptomatic/aggressive form requiring the implementation of appropriate treatment.

The complex and specific biology of CLL, based on intimate crosstalk between leukemic B cells and non-malignant T cells, undoubtedly underlies the compromised and disappointing efficacy of some therapeutic regimens (e.g., immunomodulators, immune checkpoint inhibitors, chimeric antigen receptor T (CAR-T) cell therapy) and supports CLL progression. Increasing evidence suggests that T cells not only participate in the antitumor response but also activate signals promoting the development of CLL clones in LNs [4]. More interestingly, CLL clones are highly dependent on trophic signals provided by T cells, which protect malignant B cells from apoptosis and promote their survival and proliferation [5]. In fact, T cells are able to activate CLL cells through the B cell receptor (BCR), CD40, and toll-like receptors (TLRs), thereby promoting B cell growth and anti-apoptotic pathways in CLL cells [6]. On the other hand, this intricate crosstalk with CLL cells causes phenotypic and functional defects in T cells in the TME, which are, to a large extent, CLL-specific. Functional exhaustion or anergy of CLL T cells leads to impaired activation, inhibition of effector T cell proliferation, and attenuation of cytotoxic functions, thus allowing leukemic B cells to evade immune surveillance and promoting CLL progression [7].

It is worth noting that therapeutic options for CLL have made significant progress in recent years as a result of the introduction of new drugs, such as Bruton’s tyrosine kinase inhibitors (BTKis) and BCL-2 inhibitors (BCL-2is), which interfere with survival and apoptotic signals, thereby leading to regression of malignant B cell accumulation [7]. Targeted therapy has dramatically amended the clinical response and prognosis, greatly improving progression-free survival (PFS), overall response rate (ORR), complete remission (CR) and overall survival (OS) [8]. That notwithstanding, their prolonged and repeated use is associated with the development of CLL cell resistance due to mutations acquired in the presence of strong supportive signals from the TME [4,9]. Remarkably, novel targeted therapies for CLL, such as autologous T cell-based therapies or novel tyrosine kinase inhibitors, not only influence malignant cells but also target other immune cells in the TME, primarily T cells. Therefore, when considering different therapeutic options for CLL, the implications for the T cell compartment should be acknowledged. Clarifying the role of non-malignant T cells in CLL immunobiology and disease progression in the context of CLL immunotherapy is of clinical importance.

## 2 Alterations in CLL-Associated T Cell Compartment

Although CLL is a malignancy of B cells, the T cell compartment displays several alterations in phenotype, persistence, and effector functions that are responsible for the disappointing clinical response to T cell-based therapeutic approaches (Table 1).

**Table 1:** Association of CLL-related T cell dysfunction with clinical characteristics.

T Cell Defect	Clinical Feature	References
Increased T cell counts	Correlates with progression	Matutes et al. [10] Elston et al. [11] Palma et al. [12] Brusa et al. [13]
Inverted CD4/CD8 ratio	Correlates with progression	Matutes et al. [10] Herrmann et al. [14] Gonzalez-Rodriguez et al. [15]
Imbalance in T cell subsets (e.g., increased Treg/Th17)	Correlates with progression	Jadidi-Niaragh et al. [16] D’Arena et al. [17] Hanna et al. [18] Ramsay et al. [19] Zarobkiewicz et al. [20] Can et al. [21] Kang et al. [22]
	Reduces efficacy of T cell-based therapy (BiTE, CAR-T cells)	
	Reversed by PI3Ki and IMID	
Pseudo-exhaustion phenotype (chronic activation, enhanced expression of ICs)	Correlates with progression	Motta et al. [23] Riches et al. [24] Palma et al. [12] Brusa et al. [13] Taghiloo et al. [25] Jiménez et al. [26] Kang et al. [22] Borogovac et al. [27] Robak et al. [8]
	Reduces the proliferative and cytotoxic capacity of T cells	
	Reduces efficacy of T cell-based therapy (BiTE, CAR-T cells)	
	Reversed by BCL-2i, BTKi, IMID, or ICI (when combined with BTKi)	
Impaired immune synapse formation	Reduces proliferative and cytotoxic capacity of T cells	Ramsay et al. [19] Zou et al. [28] Kabanova et al. [29] Kang et al. [22] Maffei et al. [30]
	Reduces efficacy of T cell-based therapy (BiTE, CAR-T cells)	
	Reversed by IMID and BTKi	

The functional similarities found between T cells isolated from patients with chronic viral infections and those affected by CLL suggest that persistent stimulation and antigen selection play an essential role in shaping the T cell repertoire and functionality in patients with CLL. In this review, regarding T cell dysfunctions, we focus on conventional T cells, excluding γδT cells, as well as mucosa-associated immature T (MAIT) cells and innate natural killer (iNK) T cells; however, the repertoire and prevalence of γδT cell subsets, as well as their association with CLL, are also briefly discussed.

### 2.1 Abnormalities in T Cell Receptor Repertoires

#### 2.1.1 Antigen-Driven TCR Repertoire Selection in CLL

Taking into consideration that the T cell receptor (TCR) gene repertoire in CLL is antigen selected [31], among the epitopes probably implicated in T cell selection in CLL, the most prominent roles have been assigned to: (i) the same/common antigens involved in the selection of leukemic cells [32], (ii) leukemic cell-derived antigens arising from CLL-related genomic aberrations [33,34,35], or (iii) clonotypic BCR Ig-derived epitopes [36,37].

#### 2.1.2 Diversity of αβ and γδ T Cells

The TCR/CD3 complex mediates antigen recognition and subsequent T cell activation through variable αβ or γδ TCR chains and invariant CD3 signaling subunits [38,39,40]. Antigen-driven stimulation may lead to clonal expansion of T cells sharing identical or highly similar hypervariable complementarity-determining region 3 (CDR3). αβT cells predominate in human peripheral blood and include both conventional and unconventional subsets [40,41,42,43]. Conventional αβT cells interact with short peptides presented by histocompatibility complex (MHC) class I and II together with the co-receptor CD4 or CD8 reviewed in [40,42,43], while unconventional αβT populations can recognize lipid-based antigens and small molecule metabolites presented by the MHC class I-like CD1 family and MHC class I-related protein 1 (MR1) [42,43,44,45]. Unlike αβT cells, γδT cells represent a small subset accounting for 1–5% of the total T cells in human PB [20,46]. Human γδT cells constitute a heterogeneous group [47]. As a result of TCR rearrangement, they contain one of three δ chains: δ1, δ2, or δ3, and one of six γ chains: γ2, γ3, γ4, γ5, γ8, or γ9 [48]. According to the variable fragment of the TCR δ chain, human γδT cells are divided into Vδ1, Vδ2, Vδ3, Vδ4, and Vδ5 [49,50]. It has been documented that the Vδ2 chain mostly couples with the Vγ9 chain making Vγ9Vδ2T cells, which represent the majority (up to 95%) of human circulating γδT cells [46,51]. γδT cells recognize various evolutionarily conserved antigens, including peptides, viral glycoproteins, bacterial superantigens, stress-induced phosphoantigens (small non-protein molecules containing phosphate residues), and lipids [52,53]. Most γδT cells recognize antigens independently of their presentation by MHC molecules [46]; however, up to 20% of γδT cells may interact with MHC molecules or MHC-like molecules [53,54,55,56,57]. Numerous studies indicate that γδT cells may undergo transformation into professional antigen-presenting cells (APCs), termed γδT-APCs, capable of inducing αβT CD4^+^ cell responses, as well as processing exogenous soluble proteins and presenting peptide-MHC I complexes to antigen-specific αβT CD8^+^ cells [46,58,59,60]. Furthermore, it has been demonstrated that γδT-APCs can trigger naïve αβT CD8^+^ cell proliferation and induce effector cell generation [60]. The ability of γδT cells to receive signals from and transmit signals to other immune cells, including B lymphocytes, dendritic cells, macrophages, NK cells, and αβT cells, makes these cells an integral part of both innate and adaptive immunity [46].

#### 2.1.3 Oligoclonal αβ T Cell Expansion in CLL

The first evidence of T cell oligoclonal expansion in CLL was identified by the use of a Southern blot in 1990 [61]. Based on Southern blot analysis [61], flow cytometry [62,63,64], and spectra-typing analysis [65,66], it has been demonstrated that the αβTCR repertoires of CD4 and CD8 T cells in CLL exhibit decreased diversity and skewed clonal expansion. Since clonal expansion of T cells occurs in parallel with an increase in the number of clonal B cells, it is suggested that selected T cell clones undergo expansion in response to tumor-specific antigens [65]. Expanded T cell clones in patients with CLL persist over time, and T cell expansion correlates with the tumor load, which may indicate that expanded T cell clones contain tumor-specific T cell populations [65,67].

#### 2.1.4 Phenotypic Alterations of Circulating γδ T Cells in CLL

Regarding circulating γδT cells in patients with CLL and their association with disease stage and progression, the literature is inconsistent. Nunes et al. [68] and Bartkowiak et al. [69] demonstrated increased percentages of γδT cells in the PB of patients with CLL compared with healthy controls. In contrast to these reports, Zarobkiewicz et al. [70] did not observe a significant difference in the frequency of total circulating γδT cells between patients with CLL and healthy individuals. Instead, the authors noted a significant higher percentage of γδT cells in zeta-associated protein-70 (ZAP-70)-negative patients compared to ZAP-70-positive. Flow cytometric analysis of the expression of the cytoxicity-related receptors: CD16, CD56, CD57, CD69, and lymphocyte activation gene 3 (LAG-3) revealed their altered expression on the circulating γδT cells of patients with CLL [71]. Zarobkiewicz et al. [71] observed higher expressions of both CD56 and LAG-3 receptors, lower expression of CD16, and no difference in CD69 or CD57 expression. Moreover, Zarobkiewicz et al. [70] noted that γδT cells exposed to monocyte-derived MDSCs (M-MDSC) expressed a significant increase in the levels of interleukin-10 (IL-10) *in vitro*. Furthermore, CD16 expression was significantly higher in high-risk patients (stages III and IV according to Rai classification) and inversely correlated with the serum level of lactate dehydrogenase (LDH). Moreover, patients with CLL with good prognostic markers, that is, ZAP-70-negative or CD38-negative, exhibited higher expression of CD69 and CD16, respectively. Interestingly, a separate analysis of γδT CD4^+^ and γδT CD8^+^ populations revealed that the percentage of γδT CD8^+^ cells was significantly higher in patients with CLL compared with those in the control group, whereas no difference was observed in the percentage of γδT CD4^+^ cells [70]. Additional differences in the frequency of γδT cells were noted after dividing these cells into dim/bright subsets based on CD3 and TCRγδ expression [70]. The percentage of the bright γδT cells was significantly higher in patients with CLL, whereas the dim γδT subsets dominated in healthy controls. Interestingly, the percentage of dim γδT was significantly higher in CD38-negative patients as well as in patients with mutated immunoglobulin heavy chain variable region (IGHV) genes compared to CD38-positive and those with unmutated *IGHV* genes.

#### 2.1.5 Vδ1 and Vδ2 T Cell Subsets in CLL: Clinical and Functional Relevance

When considering Vδ1T and Vδ2T cells separately, the data regarding frequency, as well as the association with disease stage and progression, are inconclusive. Zarobkiewicz et al. [72] observed that the percentage of Vδ1T lymphocytes was significantly increased in the PB of patients with CLL and trended down with an increase in the disease stage, although this was not significant. Poggi et al. [73] provided evidence that Vδ1T lymphocytes were significantly increased in patients with low risk (according to Rai-modified criteria) compared with healthy controls, whereas no increase in Vδ1T cells was found in most patients with intermediate-risk and in all patients with high risk (advanced disease). On the other hand, Bartkowiak et al. [74] and Simões et al. [75] observed an increase in the number of circulating Vδ1T cells in patients with CLL in advanced stages of the disease, according to both the Rai classification (stages III and IV) and the Binet classification (stages B and C). Interestingly, an increased number of Vδ1T cells in the PB was observed in patients whose CLL cells did not express CD38 and whose *IGHV* genes were mutated, whereas in patients with low levels of Vδ1T cells, the disease progressed within a year and the CLL cells displayed poor prognostic markers: the presence of CD38 and unmutated *IGHV* genes [73]. Similarly, Własiuk et al. [76] noticed a lower frequency of Vδ1T cells in patients whose CLL cells expressed CD38. Furthermore, Vδ1T cells from patients with CLL proliferate *in vitro* in response to autologous leukemic B cells [73,74], produce considerable amounts of interferon-γ (IFN-γ) and tumor necrosis factor-α (TNF-α) in response to autologous leukemic B cells [73], and usually have a cytotoxic profile through the expression of granzyme B (GzmB) [20,75]. With regard to Vδ2T cells, Zarobkiewicz et al. [72] did not observe changes in the percentage of Vδ2T cells (a small and statistically insignificant expansion of Vδ2T cells), which is in line with de Weerdt et al. [77] and Poggi et al. [73], who also noted an insignificant accumulation of Vδ2T cells. Furthermore, the level of the expression of CD226 (also known as DNAM-1), an activating receptor important to the cytotoxicity of NK and γδT cells, on Vδ2T cells was significantly lower in ZAP-70-positive patients [72]. CD226 expression on Vδ2T cells showed a moderate negative correlation with LDH levels and a positive correlation with serum immunoglobulin A (IgA) and immunoglobulin G (IgG) concentrations. Furthermore, Vδ2T cells secreted low amounts of IFN-γ and TNF-α in response to autologous leukemic B cells, displayed decreased cytotoxic potential through the expression of GzmB, and slight cytolytic activity against autologous leukemic B cells [73,77,78]. Of note, based on analysis of V region usage in TCR formation, Bartkowiak et al. [74] demonstrated that γδT cells from patients with CLL predominantly expressed a Vγ9 segment, usually linked to the Cδ1 region, while Vδ2^+^/Vγ9^+^ cells were observed as dominant in healthy donors, which may indicate that γδT lymphocyte expansion was driven by oligo- or polyclonal proliferation and may reflect a specific response against autologous tumor cells.

### 2.2 T Cell Dysfunction in CLL

#### 2.2.1 Altered CD4/CD8 T Cell Balance

More than 50 years ago, Catovsky et al. [79] were the first to demonstrate a significant increase in the absolute number of circulating T lymphocytes in untreated patients with CLL. Further studies have shown an increase in both CD4 and CD8 T lymphocyte subpopulations in early CLL stages, with preferential expansion of CD8 T cells [14,15,80,81,82]. This relatively greater increase in the CD8 T cell subset [10,11,12,13,83] leads to a significant reduction in the CD4/CD8 T cell ratio (even to values below 1) [15,68,80,84], which appears to affect the clinical outcome of CLL [14,84,85,86,87]. It is well documented that a reduced CD4/CD8 T cell ratio is associated with increasing CLL stage [14,80,87], development of overall hypogammaglobulinemia affecting all Ig classes [80,82], shorter lymphocyte doubling time [68], shorter time to first treatment [68,87], shorter OS [11,87], and shorter PFS [11,68]. Importantly, these findings were independent of other CLL prognostic markers, such as CD38, with zeta-associated protein-70 (ZAP-70), Immunoglobulin Heavy Chain Variable region (*IGHV*) mutation status, and Binet stage at diagnosis [11,68], pointing to the possibility that CD8 T cell expansion might not directly indicate tumor-specific changes in the T cell compartment but could also reflect cytomegalovirus (CMV) seropositivity [88]. Nevertheless, observations from a CLL mouse model have demonstrated that CD8 T cells are, at least in part, capable of controlling disease progression, because depletion of these cells resulted in shortened OS in mice [89]. In humans, a correlation between the ratio of CD8 T cells to CLL cells in the PB and better OS strengthens the suggestion that CD8 T cells attempt to control leukemia [15].

#### 2.2.2 Skewed T Cell Differentiation and Loss of Naïve T Cells

Advances in polychromatic flow cytometry technology allow more accurate identification and characterization of T lymphocyte activation and differentiation stages. The available data have demonstrated that accumulation of highly differentiated effector memory T cell subtypes [13,24,90,91,92], accompanied by a progressive decrease in naïve T cells (both CD4 and CD8 subsets) [68,93,94], is a hallmark of CLL. Furthermore, antigen-experienced CD8 T cells from patients with CLL were found to be skewed toward short-lived effector cells with significant attenuation of memory signatures [95]. This specific skewing of CLL-derived T cell differentiation was associated with ZAP-70 overexpression, a marker of poor prognosis [93].

#### 2.2.3 Activation and Inhibitory Receptor Expression in CLL-Associated T Cells

Further analysis has shown that circulating CLL-associated T cells (both CD4 and CD8 subsets) exhibit a distinct phenotype compared to healthy T cells [96]. CLL-derived T cells express higher levels of human leukocyte antigen (HLA)-DR, CD69, and Ki-67, which are markers of activation and proliferation, respectively [11,12]. Persistent antigen-driven activation of T cells in chronic infections or malignancies leads to exhaustion associated with a gradual loss of effector functions [97]. Accordingly, PB CD4 and CD8 T cells in CLL exhibit phenotypic and functional features of T cell exhaustion. Exhausted CLL-associated CD4 and CD8 T cells upregulate multiple inhibitory receptors, such as cytotoxic T lymphocyte antigen-4 (CTLA-4) [23,98], (LAG-3) [99], T-cell immunoreceptor with Ig and ITIM domains (TIGIT) [100,101], CD244 (also known as 2B4) [24], CD160 [24], and programmed cell death protein 1 (PD-1) [11,12,24,68]. PB CD8 T cells of patients with CLL also express the inhibitory molecules T-cell immunoglobulin mucin 3 (TIM-3) [25] and killer cell lectin-like receptor G1 (KLRG1) [90]. Our research group [98] was the first to demonstrate abnormal kinetics and increased surface and cytoplasmic CTLA-4 expression in both CD4 and CD8 subsets of CLL-associated T cells, indicative of increasing T cell-mediated immune suppression in CLL. CTLA-4 expression on T cells was correlated with poor prognostic factors, such as advanced Rai stage, unfavorable cytogenetics, unmutated *IGHV* status, and ZAP-70 expression [23,99]. A direct association between CTLA-4 expression and increased Treg proportion or low serum IgG and IgA levels was also observed, confirming profound immune dysfunction in CLL [23,99]. Catakovic et al. [100] and Arruga et al. [101] reported enrichment of CD4 and CD8 T cells expressing TIGIT in the PB of patients with CLL, primarily in those with an advanced disease stage (Rai II–IV) and *IGHV* unmutated status. Likewise, enriched PD-1^+^ CD8 T cell numbers were reported to positively associate with CLL burden [12,89].

#### 2.2.4 CD4 T Cells: Pro-Survival Support and Exhaustion-Like Phenotype

Notably, in CLL patient-derived xenograft mouse models, a pro-tumoral effect of autologous CD4 T cells, but not CD8 T cells, has been reported, as the presence of CD4 T cells was required for the proliferation of CLL cells [34,102]. Accordingly, CD4 T cell-derived cytokines/factors such as IFN-γ, IL-4, IL-21, and CD40L have been shown *in vitro* to induce proliferation or inhibit apoptosis of CLL cells [34,103,104,105,106]. Furthermore, depletion of PD-1^+^ and/or TIGIT^+^ CD4 T cells from co-cultures reduced CLL cell survival, which is indicative of the pro-survival impact of exhausted CD4 T cells on CLL cells [101]. This is in line with the notion that exhausted TIGIT^+^ CD4 T cells were found to produce higher amounts of IFN-γ, a cytokine promoting CLL cell growth *in vitro*. Moreover, the inverse correlations of both PD-1^+^HLA-DR^+^ CD4 and CD4 T cell abundance with CLL PFS appear to emphasize a pro-leukemic impact of the CD4 T cell subset [11,107].

Since the effects of prolonged stimulation on the phenotype, differentiation, and function of CD4^+^ T cells are not yet fully understood, and the available reports are often contradictory [108], it remains an open question whether CD4^+^ T cells become exhausted in the course of chronic infections and malignancy. Bozorgmehr et al. [109] detected that the frequencies of PB CD4^+^ T cells expressing CD160, CD244 or PD-1 were significantly higher in patients with CLL compared to healthy controls. Since all these molecules are considered exhaustion markers [108,110], this finding may indicate exhaustion of PB CD4^+^ T cells. In addition, the percentages of PB CD160^+^CD4^+^ T cells expressing IL-2, IFN-γ, or TNF-α were significantly lower compared to the frequencies of CD160-negative counterparts [109]. Similarly, the frequencies of PB TIGIT^+^CD4^+^ T cells expressing IFN-γ or TNF-α were significantly lower compared to TIGIT-negative counterparts. Of note, the frequencies of both CD4^+^TNF-α^+^ and CD4^+^IFN-γ^+^ cells were significantly lower in effector (Teff), effector memory (Tem), and central memory (Tcm) CD160-positive cells compared to CD160-negative counterparts. Perry et al. [111], Pulte et al. [112], and Zaki et al. [113] detected a significantly higher percentage of PB CD39^+^CD4^+^ T cells in patients with CLL compared to healthy controls. The high expression of CD39 is a marker of terminally differentiated exhausted (Texh-term) cells [108,110,114]. What is more, the highest frequency of CD39^+^CD4^+^ T cells was noted in patients with CLL in stages III and IV of the disease according to the Rai classification [111,112,113]. Moreover, the percentage of CD39^+^CD4^+^ T cells was significantly higher in both ZAP-70-positive [111,113] and CD38-positive [113] patients, as well as in patients with an unmutated *IGHV* gene rearrangement [111], compared to those who were ZAP-70-negative or CD38-negative and to those who had a mutated *IGHV* gene. Of note, the frequencies of CD39^+^CD4^+^ T cells and PD-1^+^CD4^+^ T cells were significantly higher in bone marrow (BM) than in PB [109,111].

#### 2.2.5 Peripheral CD8 T Cells: Exhaustion versus Pseudo-Exhaustion

In addition to augmented expression of inhibitory receptors, exhausted CD8 T cells have been functionally characterized by defective immune synapse formation, reduced cytotoxicity, gradual loss of proliferative capacity, and diminished cytokine secretion, such as IL-2, IFN-γ, and tumor necrosis factor α (TNF-α), as originally described in viral infections and several malignancies [97]. In 2013, Riches et al. [24] demonstrated that PB CD8^+^ T cells exhibit reduced proliferative and cytolytic activity but normal IL-2 expression, as well as increased production of both IFN-γ and TNF-α despite the increased expression of the inhibitory molecules PD-1, CD160, and CD244. What is more, Riches et al. [24] detected a subset of CD8^+^ T cells with high expression of B-lymphocyte-induced maturation protein 1 (BLIMP-1), a transcriptional factor playing an important role in driving CD8^+^ T cell exhaustion in chronic viral infections, which was expanded in the PB of patients with CLL. Because T cell exhaustion is a hierarchical process in which proliferation and cytolytic activity are lost first [97], the findings of Riches et al. [24] initiated a discussion about the “pseudo-exhaustion” of CD8^+^ T cells in patients with CLL. In 2018, Hanna et al. [89] noted a significant increase in the absolute numbers of CD8^+^ T cells in the PB of patients with CLL compared to healthy controls caused by expansion of the absolute numbers of both Teff and Tem CD8^+^ T cells, which positively correlated with disease development. The authors observed significantly higher absolute numbers of CD8^+^ T cells expressing PD-1 in the PB of patients with CLL than in healthy controls, which positively correlated with disease development. Moreover, the levels of PD-1 expression were significantly higher in Teff and Tem CD8^+^ T cells. In addition, the frequencies of PB CD8^+^ T cells expressing IFN-γ, TNF-α, but not IL-2, in patients with CLL were significantly higher compared to the frequencies of the corresponding cells in healthy controls [89]. Of note, Hanna et al. [89] showed that PB CD8^+^ T cells, particularly the Teff and Tem subsets, from patients with CLL exhibited higher degranulation capacity (as determined by CD107a expression) as well as cytotoxic activity via GzmB expression than PB CD8^+^ T cells from healthy controls.

#### 2.2.6 Peripheral Blood versus Lymph Node T Cell Profiles

What is more, the authors observed a distinct composition of CD8^+^ T cell subsets in the PB and LNs of patients with CLL [89]. The percentage of the Tem subset was significantly higher in LNs compared to PB, while the Teff subpopulation was significantly lower in LNs than in PB. Interestingly, subsets of terminally differentiated effector (Temra) CD8^+^ T cells were particularly low in the LNs, which is in line with published reports on preferential Temra presence in blood, spleen, BM, and lung [115,116,117,118]. Notably, CD69 and PD-1 expression levels on both CD8^+^ Teff and CD8^+^ Tem cells were significantly higher in LNs than in PB, so the increased expression of these molecules was independent of the difference in CD8 T cell subpopulation composition between LNs and PB. Functional studies have shown that LNs and PB differed not only in terms of the composition of CD8^+^ T cell subsets, but also in the functional capacities of these cells. Hanna et al. [89] detected that the frequencies of both cytotoxic CD8^+^T cells and CD8^+^ T cells expressing TNF-α were significantly lower in LNs compared to PB, and CD8^+^ T cells derived from LNs showed a tendency for lower IFN-γ expression and comparable IL-2 expression. Moreover, the frequencies of cytotoxic PD-1^+^CD8^+^ T cells, PD-1^+^CD8^+^ T cells expressing TNF-α, as well as CD8^+^ Tem subsets expressing IL-2 were significantly lower in LNs than in the PB. Similar to Hanna et al. [89], de Weerdt et al. [2] demonstrated a significant difference in the immune composition between LNs and PB in patients with CLL. The authors detected a larger proportion of Tcm cells in CD8^+^ T cells, a lower percentage of CD8^+^ Teff cells, and a higher frequency of CD8^+^ T cells expressing PD-1 in LNs than in PB [2]. Furthermore, the frequencies of total αβT cells, CLL supportive Tfh cells, and suppressive Tregs were significantly higher in LNs than in PB [2]. It is worth noting that in contrast to αβT cells, the percentages of both Vδ1 and Vδ2 T cells were more than fourfold lower in LNs than in PB, although a statistically significant difference was observed only in the case of Vδ1 T cells. The relatively low frequencies of Vδ1 and Vδ2 T cells, which exhibit cytotoxic activity toward CLL cells [20,75,77,119], as well as CD8^+^ T cells, and the increased percentages of CD8^+^PD-1^+^ T cells, Tregs, and Tfh cells in LNs indicate an immunosuppressive profile in the LNs of patients with CLL. All these observations indicate that cellular interaction in secondary lymphoid organs (SLO) leads to persistent activation of CD8^+^ T cells and accumulation of functionally exhausted T cells in LNs of patients with CLL [89].

#### 2.2.7 Trafficking-Associated and Molecular Features of T Cell Exhaustion

Since a feature of CD8^+^ Tcm cells is their ability to recirculate between blood, the T cell zones of SLO, and lymph [120], and the ability of CD8^+^ Tem cells to recirculate is controversial [110,120,121], it cannot be excluded that the exhaustion phenotype observed in LNs and the pseudo-exhaustion phenotype identified in PB may reflect dynamic transitions associated with tissue trafficking. Bozorgmehr et al. [122] investigated the migration capacity of PB CD8^+^ T cells expressing CD26. CD26 (also known as dipeptidyl peptidase 4, DPP4) is a transmembrane glycoprotein which plays a role in many cellular processes [123], including modulation of T cell activation and proliferation as well as T cell trafficking by modulating chemokines and the tendency for binding to extracellular matrix molecules and endothelial cells [124]. Based on an analysis of the expression of specific homing receptors involved in cell migration, such as C-C Chemokine Receptor 5 (CCR5), C-C Chemokine Receptor 6 (CCR6), C-C Chemokine Receptor 7 (CCR7), β7 integrin, and the common lymphocyte antigen (CLA), on CD26^+^CD8^+^ T cells, Bozorgmehr et al. [122] showed their higher migration capacity in patients with CLL, which, according to the authors, may support their trafficking to the LNs and inflamed organs or TME.

On the other hand, according to the latest knowledge, exhausted CD8^+^ T cells are defined by a distinct transcriptional and epigenetic program compared to naïve, Teff, and high-quality memory (Tem) cells [114]. It was detected that CD8^+^ Texh cells arise from memory-precursor CD8^+^ (CD8^+^ Tmp) cells, which arise as a result of the differentiation of naïve CD8^+^ T cells during acute infections [108,110,114]. During chronic infections and cancer, Tmp cells adopt a branched differentiation paradigm and differentiate into progenitor exhausted (Texh-prog) cells (also known as precursor exhausted cells, Tpex), which can self-renew in response to persisting antigens and differentiate into effector-like exhausted (Texh-eff) cells and terminally differentiated exhausted (Texh-term) cells [108,110,114]. Bozorgmehr et al. [109] detected that the frequencies of PB CD8^+^ T cells expressing CD160, CD244, or PD-1 were significantly higher in patients with CLL compared to healthy controls. In addition, the percentages of both CD160^+^CD8^+^ T cells and expressed IL-2, IFN-γ or TNF-α were significantly lower compared to frequencies of CD160-CD8^+^ T cells expressing corresponding cytokines [109]. Of note, the frequencies of both CD8^+^TNF-α^+^ and CD8^+^IFN-γ^+^ cells were significantly lower in effector and effector memory CD160-cells compared to corresponding subsets of CD160^+^ cells. Furthermore, the frequencies of CD160-CD8^+^ T cells expressing perforin or co-expressing perforin and GzmB, whose coordinated action is necessary for optimal cytotoxicity, were significantly lower compared with their positive counterparts. Interestingly, the authors [109] detected a significantly higher frequency of CD244^+^CD8^+^ T cells expressing CD107a than its negative counterparts. Moreover, Pulte et al. [112] detected a significantly higher frequency of CD39^+^CD8^+^ T cells in the PB of patients with CLL compared to healthy controls. Unfortunately, to our knowledge, there is no information on transcription factor thymocyte selection-associated high-mobility group box (TOX) expression in T cells in PB as well as in the LNs of patients with CLL. Since the development of T cell exhaustion is coordinated by this transcription factor and all Texh subsets are TOX-positive, information on its expression in both the PB and LNs, as well as the relationship between its expression and T cell functionality, would be helpful in understanding the phenomenon of exhaustion and pseudo-exhaustion in CLL.

#### 2.2.8 Functional Impairment and Anti-Leukemic Potential of CD8 T Cells

It is worth noting that functional abnormalities resulting from exhaustion were observed in T cells, regardless of CMV positivity, thus indicating a CLL-specific T cell response. Although the available data detail phenotypic analyses of the CLL-derived CD8 T cell compartment, its exact role in disease development and progression remains undefined. Similar to CD4 T cells, CLL CD8 T cells were shown to form a defective immune synapse with lower F-actin accumulation after superantigen stimulation of CLL cells [37]. In addition, further analyses revealed altered gene expression associated with cytotoxicity pathways and reduced proliferative capacity in CLL-derived CD8 T cells [24,125]. On these grounds, the functional exhaustion of CLL CD8 T cells could contribute to inadequate leukemic control. That notwithstanding, recent studies based on the enrichment of clonally expanded CD8 T cells in a CLL mouse model, together with two-year persistence of the major clonotypes in CLL patients, have strengthened the possibility of the active involvement of CLL CD8 T cells in anti-leukemia immunity [64,65,67]. This hypothesis was further supported by a study showing an association of CD8 T cells with the longer survival of leukemic mice [89].

### 2.3 Imbalance of CD4 T Cell Subsets in CLL

#### 2.3.1 CD4 T Cell Subset Diversity and Relevance in CLL

**Figure 1:**
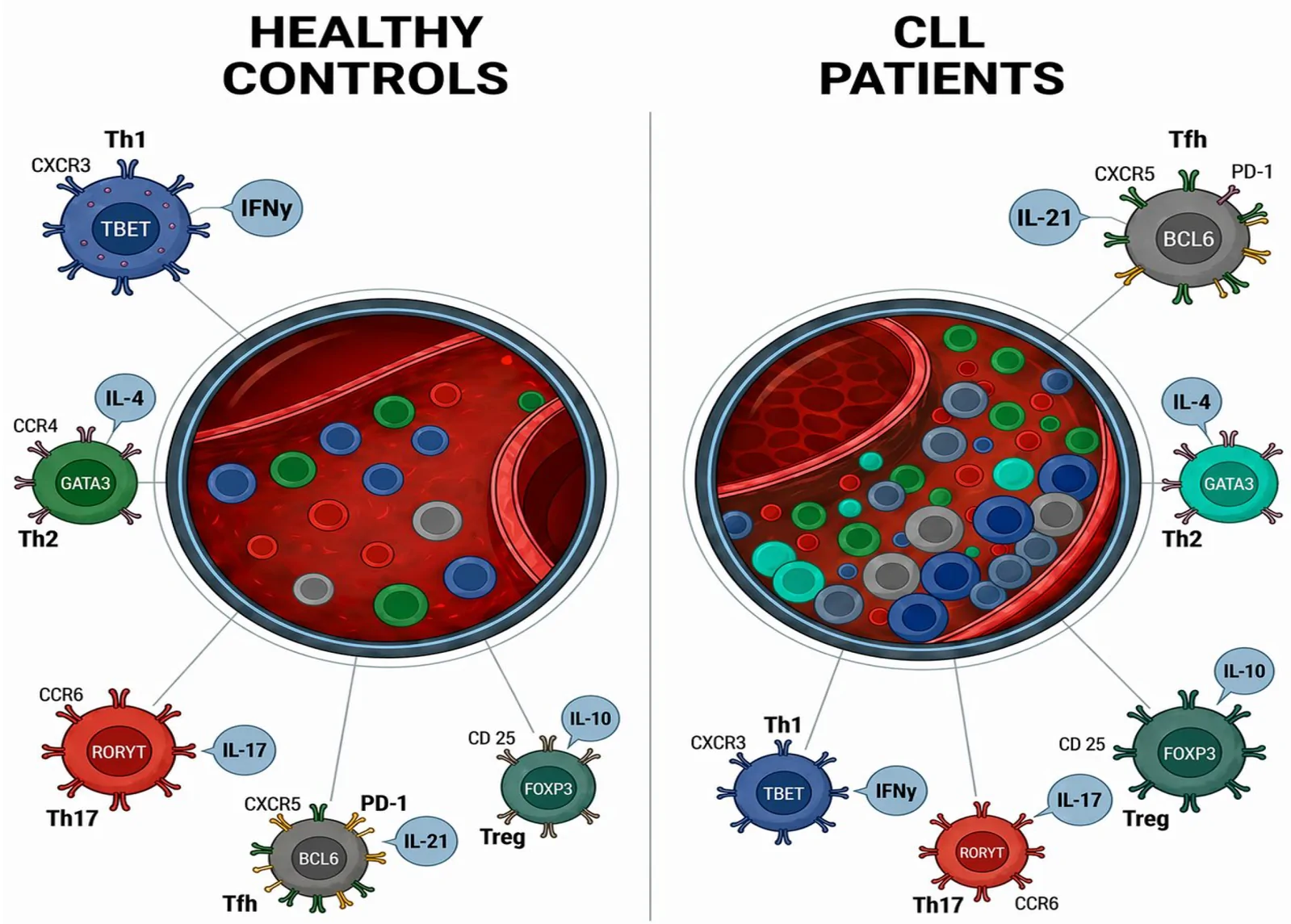
Phenotypes of CD4^+^ T cell subpopulations. Based on the expression of distinct surface markers: C-X-C chemokine receptor 3 (CXCR3) [126,127] CCR4 [126], CCR6 [126], C-X-C chemokine receptor 5 (CXCR5) [127], PD-1 [127], and CD25 [128]; cytokines: IFN-γ [126,127,129], IL-4 [126,127,129], IL-10 [128,129], IL-17 [126,127,129], and IL-21 [127,129]; and transcription factors: T-box transcription factor 21 (TBET) [127], GATA binding protein 3 (GATA3) [127], retinoic acid receptor-related orphan receptor γ (RORγT) [127], BCL6 [127], and Forkhead box protein 3 (FoxP3) [128], CD4^+^ T cells were divided into Th1, Th2, Treg, interleukin 17-secreting T cells (Th17), and Tfh cell subsets.

Since the proper balance between different T cell subpopulations is crucial for the immune response in cancer, many researchers have focused on studying the CD4 T cell imbalance in CLL and the disappointing clinical response to immunotherapeutic approaches engaging T cells. Improvements in flow cytometry methods, along with the increasing availability of phenotypic markers, have greatly expanded the possibilities for more detailed characterization of the phenotype and function of distinct T cell subpopulations in humans, as well as their differentiation pathways and role in immune responses. Based on the expression of distinct surface markers, transcription factors, and cytokines, CD4^+^ T cells were divided into T helper 1 (Th1), T helper 2 (Th2), Treg, interleukin 17-secreting T cells (Th17), and Tfh cell subsets (Fig. 1).

#### 2.3.2 Th1/Th2 Polarization in CLL

Available studies have shown an increase in the absolute number and frequencies of both IFN-γ-expressing Th1 and IL-4-producing Th2 subsets in patients with CLL compared to healthy controls [12,94]. However, controversial reports describing the dominance of Th1 T cells or Th2 T cells in CLL have also been reported. Based on the analysis of the expression profile of the genes involved in cell differentiation, Görgün et al. [125] postulated the dominance of Th2 T cells in the PB of patients with CLL. The authors observed decreased expression in a number of genes in the Ras-dependent JNK and p38 MAPK pathways, the differential profile of the expression of the genes involved in cytoskeleton formation and vesicle trafficking, as well as an increase in the expression of the genes coding actin cytoskeleton-associated proteins playing a role in cell migration/motility or cytokine production/secretory functions by controlling actin polymerization; which may lead to impairing subsequent CD4 differentiation into Th1 cells. On the other hand, cytometric analysis of the expression of CD3 and CD4 receptors, as well as IL-4 and IFNγ expression, indicated the dominance of Th1 T cells in the PB of patients with CLL [130], however, a shift Th2 T cells during disease progression was observed [130,131]. Similarly, based on cytometric analysis of the expression of surface markers, such as CXCR3, CCR6, Roessner et al. [132] demonstrated a significantly higher percentage of Th1 T cells as well as counts per μL blood in patients with CLL compared to healthy controls, whereas the percentages of Th2 T cells did not differ significantly. Analyzing the expression of the same surface markers, Palma et al. [12] noticed that patients with both non-progressive and progressive untreated CLL classified according to the modified Rai stage as defined by Hallek et al. [133] had a higher number of Th1 T cells compared to healthy controls, while higher numbers of Th2 T cells were observed in patients with non-progressive CLL. Moreover, CD4^+^ T cells of CLL patients expressed more TBET in comparison to healthy controls, whereas no difference in the expression of GATA3 was evident. In addition, cytometric analysis of the expression of both IL-4 and IFN-γ indicated that CD4^+^ T cells of CLL patients expressed IFN-γ to a higher extent in comparison to healthy controls, whereas no difference in IL-4 production was seen [132]. In addition, Roessner et al. [132] observed that the Th1 frequency remained relatively stable in CLL patients over a five-year period. Furthermore, the authors presented evidence of Th1 T cell accumulation in the Eμ-TCL1 CLL mouse model. Those studies confirmed the significance of IFN-γ-secreting T cells for CLL biology.

#### 2.3.3 Expansion and Clinical Significance of Tregs

In addition, the increase in the absolute numbers and frequency of both Tregs [134,135,136,137,138] and Th17 [139,140,141,142] cells has been well documented in a CLL patient cohort. Furthermore, it has been shown that an increased frequency of Tregs positively correlates with B-cell lymphocytosis [17,143], an absolute number of CD38^+^ B cells [17], a more advanced clinical stage (Rai and Binet stages) [137,144,145], and higher levels of LDH [17], as well as unfavorable genetics [146], and could serve as an independent predictor of time to first treatment [138,145,146,147]. Interestingly, Piper et al. [137] showed that the frequency of CD4^+^FoxP3^+^ Tregs was increased in CLL patients, but only in advanced disease, with selective expansion of FoxP3-expressing cells in the CD4^+^CD25low population, whereas the number of CD4^+^CD25high FoxP3^+^ cells was unchanged.

#### 2.3.4 MDSC-Mediated Regulation of Treg Expansion

Numerous studies indicate that MDSCs promote the differentiation of CD4^+^ T cells into Tregs and stimulate their proliferation through amino acid metabolism [148,149,150,151]. MDSCs express indole amine 2,3-dioxygenase (IDO), the enzyme that degrades L-tryptophan to kynureine-based bioproducts. Because MDSCs compete with T cells for this amino acid in the microenvironment, both a decrease in L-tryptophan level in the external environment and the production of kynurenine promote, among other things, the differentiation of CD4^+^ T cells into Tregs [148,149,150,151]. MDSCs can also recruit Tregs and stimulate their proliferation by expressing and secreting immunosuppressive cytokines, such as IL-10 and TGF-β [148,149,150,151]. Based on the presence of CD15 and CD14 molecules, two subtypes of MDSCs are distinguished: polymorphonuclear MDSCs (PMN-MDSCs), which express CD15, and M-MDSCs, which express CD14 [152,153]. It has been demonstrated that the number of PMN-MDSCs positively correlated with the number of CD4^+^ Tregs (CD4^+^FoxP3 T cells) [154]. In addition, M-MDSCs of CLL patients express high levels of IDO [155,156], as well as IL-10 and TGF-β [155]. Moreover, Jitschin et al. [155] demonstrated that M-MDSCs derived from CLL inhibited T cell activation and promoted a preferential expansion of Tregs *in vitro*. Taken together with the fact that CLL may induce Tregs [157], this observation may suggest that CLL M-MDSCs are a significant enforcer of CLL-cell-mediated Treg induction, constituting bidirectional crosstalk between CLL cells, M-MDSCs, and Tregs [155].

#### 2.3.5 Th17 Cells and the Treg/Th17 Imbalance

In the case of Th17 cells, numerous studies have shown that the percentage of Th17 cells in PB significantly correlated with lower disease stages according to the Rai classification and indolent disease stages [16,140,141,142], thus suggesting an anti-leukemic impact of the Th17 subset. Jain et al. [141] have also found that Th17 cell numbers in the blood samples of CLL patients positively correlate with OS, even in advanced-stage disease. Furthermore, Hus et al. [140] have shown that the frequency of Th17 cells is correlated with lower or negative expression of CD38 and/or ZAP-70 in CLL cells. Many researchers have focused on studying the relationship between the Treg and Th17 subsets. A few studies have shown that an increased frequency of Tregs is associated with downregulation of Th17 cells and disease progression [16,17,139,158,159], and, what is more, Th17 cells are downregulated specifically by CD39^+^ Tregs [112,160]. All those studies have strongly emphasized that an imbalance in Tregs/Th17 cells in CLL patients is an immune regulator of the disease’s clinical outcome. Interestingly, Ferrer et al. [154] demonstrated that PMN-MDSCs significantly promoted differentiation of Th17 cells and the expansion of Th17 *in vitro*.

#### 2.3.6 Tfh Cells as CLL-Supportive CD4 T Cell Subsets

The Tfh subset is one of the key players providing help for B cells undergoing selection and differentiation into activated antibody-secreting cells in mammalian germinal centers [161,162]. This is another population of CD4 T cells whose numbers increase in the PB of CLL patients, and whose frequency increases with disease progression [105,106,163,164]. Based on analysis of the expression of CD4, CXCR5, CXCR3, and CCR6 molecules using flow cytometry methods, Cha et al. [105] reported that the upregulation of Tfh cells was contributed to specifically by Tfh2 (CD4^+^CXCR5^+^CXCR3^−^CCR6^−^) and Tfh17 (CD4^+^CXCR5^+^CXCR3^−^CCR6^+^) subtypes. Of note, the authors also demonstrated that the Tfh17 subtype exhibited a clearly higher frequency in patients with an *IGHV* mutation compared to unmutated cases, thus supporting the suggestion that Tfh17 expansion could be implicated in the indolent clinical course of CLL. There is growing evidence showing that the Tfh subset of CD4 T cells promotes *in vitro* CLL growth by triggering proliferation of CLL cells via CD40L ligation in combination with IL-21 secretion [106,163]. Also, the role of IL-4 produced by LN Tfh2 cells should not be underestimated, since IL-4 was shown to boost BCR signaling, thus supporting anti-apoptotic pathways and proliferative capacity in CLL cells [165,166,167,168]. More recently, IL-4 has been assigned an additional important role in the induction of IL4I1, a factor known to be an immunosuppressive metabolic immune checkpoint in CLL-associated T cells [169,170]. In fact, elevated levels of IL4I1 were found in the sera of TCL1 mice (a mouse model of CLL), which strengthens the engagement of the Tfh subset in shaping the TME in CLL as well. Furthermore, it has also been shown that silencing the *IL4I1* gene led to improvement of the anti-leukemic response of T cells and substantially reduced the tumor load in TCL AT1 mice, thereby indicating that treatment of CLL by inhibition of IL4I1 signaling in T cells could be a favorable therapeutic strategy in CLL.

**Figure 2:**
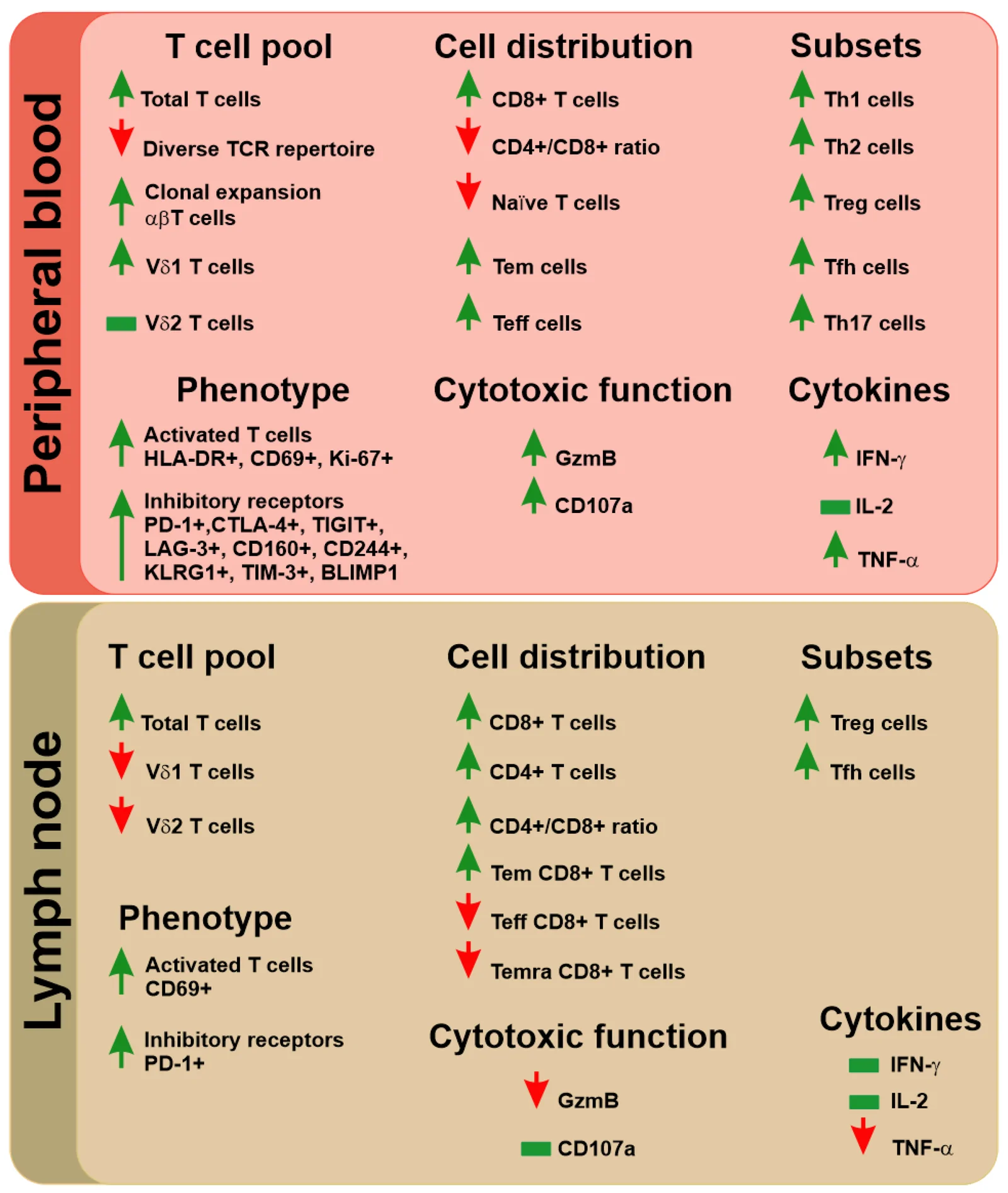
A comprehensive overview of the T-cell population and its functionality in the peripheral blood and lymph nodes of patients with CLL. The upper panel shows changes in the repertoire, number, and expression of activation markers and inhibitory receptors, as well as T-cell functionality in the peripheral blood of patients with CLL compared with healthy controls. Green and red arrows indicate increases and decreases, respectively. The green rectangles denote no statistically significant changes. The lower panel presents changes in the T-cell population between lymph nodes and peripheral blood in patients with CLL.

In summary, CLL is associated with profound quantitative and qualitative alterations in T cell populations (Fig. 2). Patients exhibit a marked expansion of total T lymphocytes, particularly CD8^+^ T cells, leading to a decreased CD4/CD8 ratio that correlates with disease progression and unfavorable clinical outcomes. Both CD4^+^ and CD8^+^ T cells show a shift from naïve toward antigen-experienced effector and effector memory subsets, alongside a reduction in naïve T cells. Clonal expansions within the TCR repertoire further indicate chronic antigen-driven selection of specific T cell populations. Within the CD4^+^ T cells, there is a clear imbalance among functional subsets. Increased frequencies of Tregs and Tfh cells are observed, both of which contribute to immunosuppression and support leukemic B cell survival. In contrast, Th17 cells are often associated with less advanced disease and may exert protective effects, highlighting the importance of the Treg/Th17 balance in CLL progression. Reports on Th1 and Th2 dominance remain inconsistent, although both subsets are frequently elevated compared to healthy controls. CD8^+^ T cells, despite their expansion, display features of chronic activation and exhaustion, including the accumulation of highly differentiated effector populations and expression of inhibitory receptors. Additionally, differences between PB and LNs indicate that the TME further shapes T cell composition, favoring exhausted and immunosuppressive subsets. Finally, increased T cell turnover, characterized by enhanced proliferation and apoptosis, reflects ongoing dysregulation of T cell homeostasis. Together, these population-level changes demonstrate that CLL reshapes the T cell compartment in a way that both impairs effective immune responses and promotes disease persistence.

### 2.4 Increased T Cell Turnover in CLL Progression

Enhancement in the T cell compartment is thought to be a unique feature of CLL [79,85,171] and could be indicative of dysregulated intrinsic mechanisms associated with T cell survival and/or apoptosis. In recent studies by our group, it has been demonstrated that the development of CLL is accompanied by increased expression of p27*^KIP1^* and cyclin D2, the proteins controlling the G0/G1 phase of the cell cycle, which may be indicative of the arrest of over 90% of PB lymphocytes in the early G1 phase and cells attempting to enter the cell cycle under *in vivo* antigen-stimulating conditions. Of interest, an increase in expression of G1-phase regulators was found to be most pronounced in the T cell subset, particularly in stable CLL. Likewise, the extent of *ex vivo* apoptosis was robustly higher in T cells, especially in progressive CLL [172]. Based on these findings, we cannot exclude that increased T cell apoptosis in aggressive CLL could be a consequence of relatively lower expression of G1 regulators when compared to the values seen in stable CLL, thus highlighting the anti-apoptotic functions of the G1 regulators examined. From our results, it also seems that differential resistance to apoptosis observed in B and T cells in CLL patients may determine the CLL clinical course. In fact, we found that PB T cell apoptosis heightened the risk of CLL progression, whereas the rate of CLL cell apoptosis was of protective significance [172]. Remarkably, given that CLL patients exhibited a marked increase in the absolute number of blood T lymphocytes at each stage of the disease, our findings on increased CLL-derived T cell apoptosis, primarily during progression, may be suggestive of the higher proliferative capacity of CLL T lymphocytes [173,174,175].

These initial findings were followed by more extensive genetic and epigenetic assays performed by our group, prompting speculation that increased proliferation of CLL T cells could result, at least in part, from aberrant intrinsic signals [175,176]. Accordingly, we found that carriers of the rs36228499CC and rs34330CC genetic variants at the *CDKN1B* and *CCND2* loci encoding the studied G1-phase regulators exhibited lower levels of the regulatory proteins p27*^KIP1^* and cyclin D2 in T cells compared to CLL patients lacking these polymorphic variants, an observation attributed to greater cell proliferative potential [175,176,177,178,179]. In line with this, CLL patients carrying the same genotypes displayed significantly shortened time to disease progression [176]. Collectively, our studies imply that increased T cell apoptosis in progressive CLL appears to be compensated by increased T cell proliferation. This is in line with the previous notion that proliferating lymphocytes in CLL are more prone to apoptosis [180]. In this regard, we hypothesize that increased T cell turnover in CLL may reflect the maintenance of a pool of T cells promoting the survival of leukemic B cells and favoring an aggressive clinical course [181]. The above studies clearly indicate the involvement of non-malignant T lymphocytes in the clinical outcome of CLL and may help identify patients with higher T cell turnover requiring caution when an immunotherapeutic approach affecting T cell division is administered. Further studies are certainly needed to confirm our preliminary observations.

**Figure 3:**
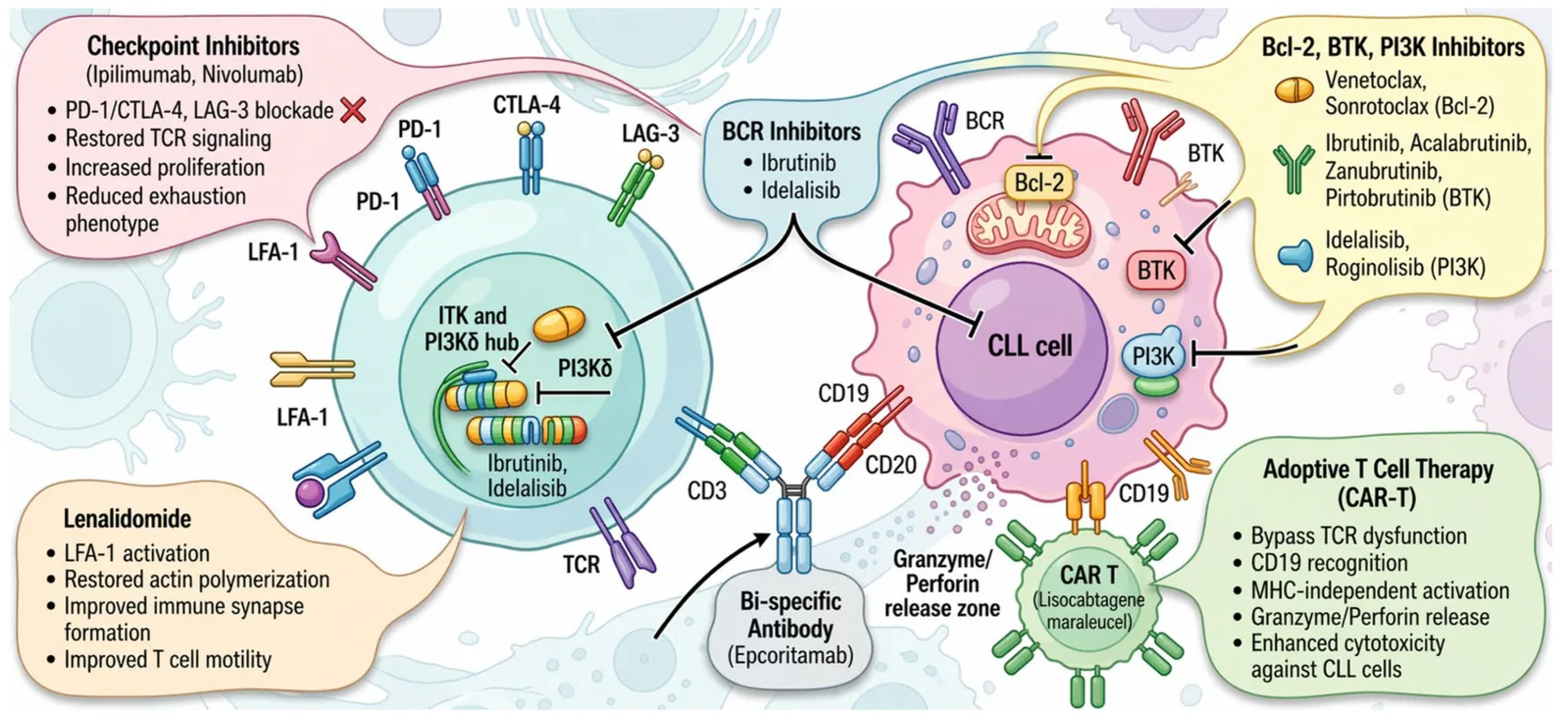
Possible mechanisms for restoring T cell functionality in CLL under different therapeutic interventions. Novel therapies target the key membrane-bound and signaling proteins in leukemic B and non-malignant T cells, including CD19, CD20, CD3, LFA-1, PD-1/PD-L1, CTLA-4, LAG-3, Bcl-2, BTK, PI3K, and ITK. Therapeutic modalities are based on monoclonal antibodies, bispecific antibodies, small-molecule inhibitors (e.g., immune checkpoint, kinase, or Bcl-2 inhibitors), and CAR-T cells.

Our findings on the intrinsic proliferation and apoptosis defects in CLL-derived T cells may have clinical implications. They indicate that certain polymorphisms in genes encoding proteins involved in cell division could be associated with the rapid progression of CLL and considered as prognostic factors for shortened PFS. At this preliminary stage, our data do not allow strong conclusions to be drawn with regard to the best therapeutic approach for patients possessing risk variants in the *CDKN1B* and *CCND2* genes. We cannot exclude that patients with reduced activity of p27*^KIP1^* and cyclin D2 in T cells determined by genetic variants of these genes would not benefit from BTKi or BCL-2i therapy due to increased apoptosis and turnover in the T cell compartment, which is of clinical risk for CLL progression. Further studies are warranted to determine whether *CDKN1B* and *CCND2* gene polymorphisms do influence the clinical efficacy of the targeted therapies currently used in CLL management, such as BTKis, BCL-2is, or CAR-T cell adoptive transfer, which were shown to reveal off-target activity within the T cell compartment apart from a direct on-target effect on CLL cells. That notwithstanding, immunotherapy approaches in CLL based on the systemic administration of drugs targeting both B and T cells should be provided with caution and personalized toward targeted options (Fig. 3).

## 3 Immunotherapy of CLL: Implications for the T Cell Compartment

Herein, we present the current view on the therapies or potentially useful investigational options for the treatment of CLL, revealing a significant impact on the T cell population. Available targeted treatments in CLL, although with highly improved clinical outcomes, remain non-curative. In recent years, immunomodulatory drugs, immune checkpoint inhibitors, adoptive T-cell therapy (including CAR-T cells), and BCR inhibitors (including BTKis) [168,182], BCL-2is, BTK degraders, PI3K inhibitors, and T cell engagers have been introduced into the clinic or are of potentially clinical relevance. Although these drugs have different mechanisms of action in the immune system, they are known to affect the T cell compartment, apart from directly affecting CLL cells (Table 2). The mechanism of action is based on restoration of the B–T cell immune synapse and reinvigorating T cell-dependent antitumor responses (Fig. 3). Given that T cells play an ambiguous role in the pathogenesis and clinical course of the disease, in-depth knowledge of the role of T cells in the development and progression of CLL is essential for proper stratification of the benefit-to-risk ratio with regard to immunotherapeutic strategies affecting the T cell compartment.

**Table 2:** Impact of the T cell-based therapies on T cell compartment.

Treatment	Association with T Cell Dysfunction	References
CAR-T cell transfer (autologous)	Reduced efficacy due to hampered function, proliferation, and exhaustion of T cells harvested from patients	Lemal and Tournilhac [183] Borogovac et al. [27] Roessner and Seiffert [168] Camerini et al. [7]
	Limited expansion and persistence capacity of CAR-T cells	
Bispecific T cell engager (e.g., blinatumomab)	Reduced efficacy due to impaired cytotoxic function and exhaustion of T cells	Kang et al. [22]
Chemoimmunotherapy (e.g., FC)	Aggravation of existing T cell immunodeficiency (fatal severe infections)	Papazoglou et al. [184]
BTK inhibitor (e.g., ibtuninib)	off-target activity in T cells	Yin et al. [185] Niemann et al. [186] Long et al. [187] Davis et al. [188] Parry et al. [189] Zou et al. [28] Fan et al. [190] Papazoglou et al. [184]
	Initial inhibition of ITK and T cell activation, then improvement of defective T cell function over time (long-term effect) with increase in T cell proliferation and reversion of exhaustion	
	Restored immune synapse formation	
	Reinvigoration of T cells before CAR-T cell transfer	
PI3K inhibitor (e.g., idelasib)	off-target activity in T cells Reduction of Treg count	Hanna et al. [18] Chellappa et al. [191]
	Aggravation of preexisting cytotoxic CD8^+^ T cell dysfunction	
BCL-2 inhibitor (e.g., venetoclax)	off-target activity in T cells	Arunachalam et al. [192] Van Bruggen et al. [193]
	Reversion of T cell exhaustion and dysfunction	
	Reinvigoration of T cells before CAR-T cell transfer	
Immunomodulatory drug (e.g., lenalidomide)	Restoration of T cell migration and synapse formation	Ramsay et al. [19] Kabanova et al. [96]
	Modulation of TME (reduction of Treg and improvement of T cell function)	
Immune checkpoint inhibitor (e.g., nivolumab, pembrolizumab)	Reduced efficacy in monotherapy (in R/R CLL and Richter’s transformation)	Ding et al. [194] Younes et al. [195] Mu et al. [196]
	Reversion of T cell exhaustion (when combined with ibrutinib)	

For many years, chemoimmunotherapy (CIT) was administered for progressive and symptomatic CLL in treatment-naïve (TN) and R/R patients [1]. This form of treatment included cytotoxic drugs (e.g., cyclophosphamide, fludarabine, bendamustine, and chlorambucil) as well as CD20-targeting monoclonal antibodies (rituximab and obinutuzumab). In the past decade, treatment based on targeted oral regimens has replaced CIT as a standard-of-care for TN CLL. Currently, frontline treatment of CLL consists of two main strategies, including continuous therapy with covalent BKTis (cBTKis) and fixed-duration regimens, which include the BCL-2i venetoclax combined with either anti-CD20 antibodies or BTKis [197,198]. The most common regimen with continuous administration of a cBTKi is used until the therapy is no longer tolerated or effective. When selecting either continuous or fixed-duration treatment, both molecular and patient factors should be taken into consideration. Continuous therapy with a single BTKi is usually dedicated to older patients, with higher-risk factors and/or competing comorbidities. On the other hand, younger patients, with lower-risk CLL may benefit from fixed-duration therapy. This time-limited venetoclax-based approach allows patients to have treatment-free intervals lasting as long as many years until new therapy is required. Remarkable, both regimens (continuous and time-limited) are safe and extraordinarily effective with very high rates of CR, including frequent undetectable minimal residual disease (uMRD) state [1,197]. Yet, therapeutic decisions on the optimal sequencing of therapies for an individual patient is complex and depends on prior treatment, the duration of the clinical response, and risk-factor status.

### 3.1 BTK Inhibitors

The administration of BTKis as a first targeted therapy for CLL opened a new era in disease management. Although CLL is still considered an incurable disease, the introduction of BCR inhibitors into therapy has significantly increased treatment options. The similarity of B and T cell kinases is a characteristic enabling BCR inhibitor cross-reactivity and modulation of T cell phenotype, persistence, and antitumor efficacy, apart from the modulation of B cell survival signals. Despite a diminishing impact of BCR inhibitors on the signal transduction of the activated BCR in CLL cells [199], they were shown to influence immunomodulatory effects in the TME [185,186,187,188,189].

Ibrutinib, an irreversible inhibitor of BTK commonly used for CLL therapy, has been demonstrated to inhibit BCR signaling, which is associated with a reduced tumor burden via a direct on-target effect in CLL cells [28]. It also leads to disruption of the TME interactions with subsequent redistribution of malignant B cells into the PB. In addition, it reveals direct off-target activity attributed to a significant impact on CD4^+^ and CD8 T cells subsets [185,186,187,188,189]. In T cells, it was found to target interleukin-2–inducible T cell–associated kinase (ITK) involved in T cell signaling and immunity [200,201]. Ibrutinib-mediated impact on ITK was linked to a shift of T cells toward a Th1 phenotype, thereby promoting the antitumor responses of T cells. In addition, clonal expansion of T cells with a broader TCR repertoire diversity after one year of therapy with ibrutinib was also reported [185]. Yet, there are contradictory results describing the influence of ibrutinib on T cell function and numbers in the PB [185,186,187]. In some studies, ibrutinib was shown to attenuate T cell activation, proliferation, and effector function upon *ex vivo* treatment [202] and in a CLL mouse model [203], and contributes to the expansion of fully competent specific T cell clones. Whereas in other studies, similarly to second-generation BTKis (acalabrutinib, zanubrutinib), ibrutinib was reported to restore T cell proliferation and immune synapse formation [28,186]. It could also reverse T cell exhaustion and inflammatory cytokine production. In addition, ibrutinib was demonstrated to affect other cells in the TME, such as macrophages and MDSCs [204]. In macrophages, it blocks BTK signals and downregulates the expression of chemokines (CXCL12, CXCL13), which are involved in CLL cell activation and drug-resistance development [186]. Administration of novel, more specific BCR inhibitors, such as acalabrutinib, a covalent inhibitor of BTK showing lower affinity for ITK, could help determine whether the observed modulation of the TME in BCR inhibitor-treated CLL patients is caused by direct inhibition of ITK or indirectly by the diminished impact of CLL cells on their surroundings and normalization of the inflammatory microenvironment [185,186,187,205,206,207,208] (Fig. 3).

Ibrutinib was the first oral covalent BTKi approved by the Food and Drug Administration (FDA) for the treatment of CLL. Based on the mechanisms underlying the binding to BTK, these agents have been classified into two types: covalent (irreversible) and non-covalent (reversible) [198]. The latter remain effective despite mutations of the C481 residue of the BTK which is a typical binding site for covalent inhibitors. cBTKis include a first-generation inhibitor (ibrutinib) approved for patients with R/R CLL (in 2014) and as a first-line treatment for TN CLL (in 2016), of which the latter was extended to be used in combination with anti-CD20 antibodies (obinutuzumab and rituximab) in frontline settings. The better tolerated second-generation cBTKis (acalabrutinib and zanubrutinib) could be administered in continuous monotherapy as first-line treatment for CLL [1,197]. Acalabrutinib was also approved as a combined therapy with obinutuzumab. In February 2026, based on the AMPLIFY trial ((NCT03836261) [209], the FDA approved acalabrutinib plus venetoclax in a fixed-duration (14-months) therapy for frontline CLL.

Remarkably, clinical trials evaluating cBTKis have confirmed superior ORR, PFS, and OS compared to clinical response of CIT (with FCR, Chl, anti-CD20) [210,211,212]. Currently, the usage of first-generation cBTKis is limited due to their toxicity (primarily cardiovascular adverse events) [213], while second-generation cBTKis exhibit improved safety profiles and comparable efficacy [214,215,216] and are preferred in high-risk CLL management [214,216]. The next generation of cBTKis, such as orelabrutnib and tirabrutinib, are still under investigation for B cell malignancies, including CLL [198]. Non-covalent BTKis (ncBTKis), such as pirobrutinib and nemtabrutinib, are next-generation agents that effectively inhibit BCR signaling even in the presence of acquired mutations in the BTK pathway, which were found to confer resistance to cBTKis [217]. These are highly promising approaches for R/R CLL patients previously treated with cBTKis and BCL-2is [218], for whom pirobrutinib received FDA approval in 2025 [218]. Of note, ibrutinib was also successfully administered for reinvigoration of T cells before CAR T-cell transfer to reach an improved clinical response [184,190].

### 3.2 BTK Degraders

BTK degraders are another agent triggering selective and rapid BTK depletion dedicated to patients with CLL, exhibiting BTKi intolerance or acquired resistance [219] These proteolysis targeting chimeras (PROTACS) are capable of inducing BTK proteolysis degradation [219]. They are currently being investigated in early clinical trials, showing favorable safety and efficacy profiles in CLL cases resistant to BTKis [219,220], thus indicating a promising approach.

### 3.3 BCL-2 Inhibitors

Inhibitors of BCL-2 target anti-apoptotic protein BCL-2, which is overexpressed in malignant B cells. The first-generation BCL-2i venetoclax disrupts the BCL-2 interaction with pro-apoptotic proteins via direct on-target activity, whereby inducing apoptosis of CLL cells [221]. Venetoclax was also shown to affect the T cell compartment, which is probably related to its off-target activity. It has been demonstrated that after receiving venetoclax, the naïve T cells of patients with CLL decreased and memory T cells increased [222]. Also, patients receiving venetoclax with obinutuzumab displayed downregulation in the levels of T cells, B cells, NK cells in PB, and reversion of T cell exhaustion with inhibition of inflammatory cytokine secretion [2,193]. Given the observed 80% ORR in phase 2 trials [223], monotherapy with venetoclax was approved by the FDA in 2016 for continuous therapy of R/R patients possessing del(17p). The most commonly used regimen is fixed-duration therapy with venetoclax in combination with CD20-targeting monoclonal antibodies [224,225]: rituximab for R/R and obinutuzumab for TN CLL approved by the FDA, respectively, in 2018 and 2019. The frontline administration of venetoclax with obinutuzumab (Ven-O) has an approved indication for TN patients with CLL with comorbidities using a one-year course of treatment. In a clinical study conducted in R/R CLL, time-limited therapy with venetoclax plus rituximab resulted in time durable remissions and superior PFS and OS in comparison to the CIT arm (bendamustine plus rituximab) [226]. In turn, the fixed-duration Ven-O therapy in TN CLL, including the high-risk subgroup with mutation in TP53, del(17p) or umIGHV, led to substantially higher PFS compared to CIT (chlorambucil plus obinutuzumab regimen). Yet, the presence of del(17p) or umIGHV in the Ven-O arm was found to be an independent prognostic factor for shortened PFS [227].

Another fully-oral fixed-duration alternative for patients with CLL is a combination of venetoclax with BTKis [210,226]. Among several clinical trials, the most studied regimen was the combination of venetoclax and ibrutinib [228], however, venetoclax in combination with other BTKis (acalabrutinib, zanubrutinib) was also evaluated; venetoclax combinations were administered to TN patients, and the majority were compared to the CIT arm, showing durable OR with significantly higher and impressive rates of uMRD, PFS, and OS, as well as lower CLL progression cases. Notably, the presence of genetic higher-risk factors significantly affected the clinical response even in the venetoclax plus ibrutinib arm. Clinical analysis of all those trials was discussed in detail in recent works [8,229]. Similarly to ibrutinib, venetoclax was showed to improve T cell fitness before CAR-T cell transfer [192]. Based on extraordinary efficacy and safety profiles observed in the AMPLIFY trial (NCT03836261), which showed superior progression-free survival (PFS) compared to standard CIT, the combinations of venetoclax plus ibrutnib or acalabrutinib were approved by the European Medicines Agency (EMA) (in 2022 and 2025, respectively) as a front-line fixed-duration treatment the European Union (EU).

### 3.4 PI3K Inhibitors

Clinically approved PI3K inhibitors, such as idelalisib, have been demonstrated to specifically reduce the immunosuppressive function and numbers of Tregs, which could be a favorable strategy in CLL for modulating the immunosuppressive TME. However, idelalisib-mediated specific Treg inhibition was shown to augment the cytotoxic activity of CD8 T cells only in solid tumors [230]. No relevant impact of idelalisib on the differentiation and effector capacity of CD8 T cells was found in TCL1 leukemic mice (expressing T-cell leukemia/lymphoma 1 (*TCL1*) gene), most likely due to inhibition of TCR signaling [18]. Therefore, the clinical efficacy of CLL therapy with idelalisib appears to be limited by increased numbers of autoimmune adverse events and infection rates [191,231,232,233,234] This effect is dependent on idelalisib-mediated reversal of Treg-mediated immune suppression and T cell imbalance of regulatory and cytotoxic subsets [18,191]. While PI3Kis (idelalisib, duvelalisib) had historically been approved by the FDA and EMA for R/R CLL with high-risk factors (del(17p)/TP53 mutations), these agents have largely been restricted to a later line treatment option due to the high toxicity (e.g., fatal infections and immune-mediated events).

### 3.5 Immunomodulatory Drugs

Defective immune synapse formation is a hallmark of CLL resulting from altered expression of the genes engaged in actin polymerization and cytoskeletal organization [19,29,235]. Proper activation of T cells requires formation of the immune synapse allowing specialized contact between the TCR and HLA-bound antigen in the presence of costimulatory signals [236,237]. It has been shown that insufficient costimulatory support mediated by CLL cells [238], a mechanism used to evade immune detection, leads to inappropriate synapse formation, thereby revealing a deleterious impact on T cell signaling and antitumor immunity [19,29,238,239]. There is growing evidence that synapse defects could be reversible by administration of immunomodulatory drugs, such as lenalidomide, that regulate cytokine expression and mobilize T cell-mediated antitumor responses [240,241,242]. Lenalidomide is an immunomodulatory drug, which differs from targeted therapeutics in that its mode of action is based on repairing the TME, but not directly killing CLL cells. CLL therapy with lenalidomide was shown to stimulate T cell migration due to activation of LFA-1, an integrin disturbed by contact with CLL cells [241]. Clinical studies with immunomodulatory drugs have demonstrated improvement of clinical responses by restoration of immune synapse formation and T cell activation and effector functions [19,243] (Fig. 3). In the area of CLL targeted therapies with BTKis and BCL-2is, its role is mostly restricted to specific high-risk scenarios or therapy within clinical trials due to toxicity concerns (e.g., tumor flare reactions, myelosuppression, fatal tumor lysis syndrome). Of note, the ORIGIN study (NCT00910910) showed that CLL monotherapy with lenalidomide led to increased mortality compared to the chlorambucil arm [244]. In the clinical trials, lenalidomide could be used in the immunotherapy designed to overcome the limitations of single-agent therapies in R/R patients with CLL. While lenalidomide does not has an approved indication for CLL in a standard therapy, research continues into its effectiveness in combination with other agents (e.g., rituximab or obinutuzumab) or as maintenance/consolidation therapy for high-risk patients, who had achieved frontline CIT or targeted therapy [245]. In some cases, administration of lenalidomide has been shown to prolong PFS (CONTINUUM trial; NCT00774345) even without a significant improvement in OS [246]. Moreover, long-term studies reported that early intervention with lenalidomide in high-risk asymptomatic patients with CLL potentially delays the initiation of treatment [247]. Therefore, the role of lenalidomide seems to be limited to combined regimens, as it is currently being investigated in clinical trials in patients with relapsed CLL (NCT02568553). This study has been evaluating the clinical efficacy and safety of the combination of blinatumomab and lenalidomide. Blinatumomab, a T cell engager which is a bispecific monoclonal antibody targeting CD3 and CD19, is a promising approach, as it exerted preclinical efficacy in CLL by inducing autologous T cell cytotoxicity against CLL cell lines from TN and R/R patients [248]. Its combination with lenalidomide appears appropriate, since chronic T cell exposure to blinatumomab could enhance T cell exhaustion [249]. In this study (NCT02568553), lenalidomide was shown to enhance the antitumor efficacy of blinatumomab by reversing T cell exhaustion (and boosting T cell activity) and help to more effectively bridge T cells to CLL cells, which led to robust CLL cell death. This study is promising and directly relevant for high-risk patients with limited options, who have relapsed after multiple intensive treatments. Lenalidomide is also being investigated in CAR-T cell options, where it is administered to temper an immunosuppression mediated by a leukemic TME and in accordance with its ability to preserve the CAR-T-CLL cell synapse [250,251].

### 3.6 Immune Checkpoint Inhibition

Research into immune checkpoint receptors and their ligands has opened a new area of treatment modalities for tumor immunotherapy. One key mechanism underlying tumor escape from immune surveillance is the engagement of immune checkpoint molecules on T cells. Accordingly, CLL cells, by overexpressing inhibitory ligands, are able to target inhibitory receptors on T cells and induce regulatory pathways [13,252]. In addition, upregulated expression of inhibitory receptors, such as PD-1, CTLA-4, BTLA, CD244, LAG-3, TIGIT, and CD160, reported on T cells from the PB and LNs of patients, could facilitate inhibitory receptor–ligand interactions [7,99]. Yet, *in vitro* reinvigoration studies with anti-TIM-3 and anti-PD-1 blocking antibodies did not markedly improve cytotoxic and proliferative ability, nor cytokine secretion, in CLL T cells [253,254]. Likewise, despite encouraging results from preclinical studies in a CLL mouse model and clinical studies in solid tumors [255,256], administration of monoclonal antibodies blocking immune checkpoints or their ligands in CLL clinical settings has been highly disappointing. Single-agent therapy of relapsed patients with a humanized anti-PD-1 antibody had no or only minimal clinical response, limited to CLL cases who developed Richter syndrome, which is a minority of CLL cases [194]. One reason for the discrepancies observed could be the different clinical stages between mice and patients enrolled in the studies, supporting the hypothesis that an immune checkpoint blockade could be a beneficial strategy for the early stages of CLL development. Another possible explanation for checkpoint blockade ineffectiveness in CLL is that patient T cells were shown to display a pseudo-exhausted phenotype with retained cytokine secretion [24], as CLL-derived T cells showed functional defects in proliferation and cytotoxicity, but produced higher amounts of IFN-γ and TNF-α and normal levels of IL-2 [24]. Also, the malfunction of checkpoint receptors on CLL-derived T cells cannot be excluded, according to indices of bidirectional or costimulatory signaling mediated by BTLA, hence triggering activation of the PI3K/AKT or NF-κB pathways, respectively, with an increase in T cell activation and proliferation [257,258]. Our most recent study performed in CLL has confirmed the above suggestion, showing enhanced proliferation and IL-4 secretion in BTLA^+^ compared to BTLA− T cells of patients with CLL [175]. A more recent consideration is associated with the finding that an exhaustion transcriptomic signature has been found in non-CLL-specific T cells in TCL1 AT mice [259], indicating that dysfunctional exhaustion of CLL T cells may not result from tumor antigen-mediated stimulation, but could be a bystander effect assigned to T cells that do not recognize malignant B cells. In consequence, CLL T cell dysfunction may exist even in the absence of tumor antigen-T cell crosstalk and is dependent on the mere presence of CLL cells. This clearly points to the possibility that CLL cells, especially during progression, are able to affect T cell responses through secretion of inhibitory cytokines (IL-9) or epigenetic reprogramming of CD8 T cell responses toward short-lived effectors, thereby bypassing inhibitory receptors [91,240]. Removal of CLL cells has been demonstrated to alleviate T cell dysfunction, thus emphasizing that T cell impairment in CLL is, at least in part, induced by CLL cells.

While checkpoint inhibitors have revolutionized the treatment of solid tumors and Hodgkin lymphoma, they have shown limited efficacy when used as single agents in B cell malignancies like CLL [194]. As of early 2026, due to limited efficacy, immune checkpoint inhibitors, such as PD-1/PD-L1 inhibitors (pembrolizumab, nivolumab) and CTLA-4 inhibitors (ipilimumab), do not have FDA approval for the treatment of CLL and are not the standard-of-care for patients with CLL. Ongoing clinical studies are investigating the combination of checkpoint inhibitors with BTKis (like ibrutinib) or BCL-2is (like venetoclax) to overcome the T cell exhaustion commonly seen in patients with CLL [195,196]. Specifically, *in vitro* and *in vivo* administration of venetoclax, a BCL-2i, due to the reduction of the leukemic burden, led to the partial rescue of T cell metabolic dysfunctions with the reversal of T cell exhaustion features and Treg expansion [2,193,260]. Of interest, treatment of patients with advanced-stage CLL based on checkpoint inhibitors combined with BTKis (ibrutinib) showed promising results with a significant improvement in the clinical response rate [195]. These results correspond with data obtained from the TCL1 AT mouse model, where combined treatment approaches resulted in almost complete eradication of CLL cells in lymphoid tissues, in contrast to ibrutinib or a checkpoint inhibitor administered alone [203].

Remarkably, recent observations from trials have undoubtedly shown that the clinical response to checkpoint blockade in CLL is significantly poorer than that observed in solid tumors. Furthermore, for a subset of patients, some immunotherapy methods interfering with T cells may even pose a risk of earlier disease progression, as we demonstrated in our previous study [261]. Given that systemic administration of checkpoint inhibitors in B cell malignancies, such as CLL and multiple myeloma, enables targeting of inhibitory receptors on both T cells and B cells, this form of immunotherapy could be of clinical risk; in fact, we observed that checkpoint inhibitors could affect the time to disease progression in patients with inappropriate PD-1/CTLA-4 levels depending on the disease stage and/or treatment status [261,262]. In addition, recent evidence from our group supported epigenetic modification of BTLA checkpoint protein expression in CLL cells [174]. We have also reported that BTLA unfavorably promotes T cell proliferation and IL-4 secretion in T cells, a finding consistent with BTLA malfunction observed by others [257,258]. Since epigenomic characterization of CLL-derived T cells remains limited, assessment of the role of the epigenetic regulation of inhibitory receptors in T cells is of clinical relevance. Addressing this question, we demonstrated that miR-155-5p silencing does not increase BTLA molecule expression in CLL-derived T cells and would be a safe strategy in CLL patients [175]. As we recently showed [174], this form of therapy would benefit a subset of CLL patients with impaired BTLA levels on B cells in order to normalize CLL cell activation.

### 3.7 Adoptive T Cell Therapy

Adoptive T cell therapy is a promising field of CLL management. Its protocol is based on the infusion of genetically modified autologous T cells with specificity for the antigens present on malignant B cells [27]. This increased T cell specificity against CLL cells is achieved by the *ex vivo* genetic introduction of a CAR with affinity for CD19, an antigen used as a selective B cell target. Engagement of a CAR-T cell with CD19 on B cells results in T cell activation in an MHC-independent manner and redirects cytotoxic responses toward CLL cells [263]. Although increasing the specificity of T cells for malignant cells by CAR-T cell therapy has improved immunotherapeutic approaches and clinical response in B acute lymphoblastic leukemia, the results of adoptive T cell therapy in CLL have not been as spectacular: CR was seen in only about 21–29% of R/R patients [264,265]. Furthermore, a high rate of patients experiencing symptoms of cytokine release syndrome as side effects was also reported, which significantly weakens the clinical benefits for this form of CLL immunotherapy [264,265].

The CLL-associated T cell defects described above, primarily an exhausted and antigen-experienced effector phenotype, highlight the key obstacle in effective CAR-T cell therapy for CLL. In order to overcome these limitations and induce more vigorous antitumor properties, several modifications during CAR-T cell production, such as constitutive expression of CD40L, PD-1 blockade, metabolic reprogramming, and administration of cord-derived CAR-transduced NK cells [266,267,268,269], have recently been presented in investigational studies. The latter approach is highly appreciated, given that no full HLA match between donor and host is necessary, thereby bypassing the requirement to use autologous cells for CAR transduction [269]. Also, available data showed that stimulating CLL cells via CD40L-expressing CAR-T cells could improve CLL cell capacity for antigen presentation, thereby reinvigorating the antitumor efficacy of autologous T cell-based therapy [239,266]. Accordingly, a preclinical model of B cell lymphoma using CAR-T cells with constitutive CD40L expression confirmed the above suggestion [266]. Recently, it has been reported that *ex vivo* CAR-T cell manufacturing triggers PI3K signaling as well, resulting in maintenance of a more terminally differentiated phenotype of CAR-modified T cells with suboptimal *in vivo* persistence [270]. To bypass this limitation, inhibition of the PI3K–mTOR signaling axis with a PI3K inhibitor (duvelisib) has been introduced in CLL, leading to the induction of more stable numbers of CAR-T cells in a less differentiated state, with augmented antitumor functions [271]. Similar results were noted in studies with AKT, another kinase of the PI3K pathway, inhibition of which also contributed to increased cytotoxic function and persistence of CAR-T cells [272], thereby giving hope for an improvement of CLL the patient clinical response to CAR-T cell therapy.

Given that autologous CAR-T cell therapy is already personalized, a major challenge for effective CAR transduction is designing T cells highly specific toward CLL cells. Therefore, it is of clinical significance to identify and characterize anti-leukemic T cell clones together with respective tumor-derived antigens expressed on CLL cells as potential effective targets for CAR-T cells. Kowalewski et al. [273] identified tumor-associated T cell antigens exclusively and robustly expressed in the HLA ligandome of CLL cells, correlating with specific immune recognition by patient T cells regardless of standard chemo- and/or immunotherapy. Also, HLA-presented antigenome studies proposed other neoepitopes derived from CLL-associated genomic aberrations that have been found exclusively on CLL cells as proper targets for CAR-T cell products [32,35,274].

Another concern that should be acknowledged when considering adoptive T cell therapy is generation of the CAR-T cell product exhibiting a phenotype associated with appropriate T cell functionality and persistence. This is in line with the observation that differences in T cell phenotype and subset distribution prior to CAR-T cell generation can significantly impact the features and activity of CAR-T products, thus determining the clinical outcome of this strategy [275,276]. For successful adoptive cell therapy of CLL, only expanded and persistent T cells transferred during the CAR-T cell approach are able to develop long-term tumor immunity [275]. There is evidence that long-term persistence is a functional characteristic assigned to less differentiated (central) memory T cells. This contrasts with late-stage differentiated effector CD8 T cells that rapidly undergo apoptosis following CAR-T cell transfer [91,95], thus dampening the efficacy of this adoptive treatment strategy. Comprehensive analysis of the determinants of the clinical response to CAR-T cell therapy revealed that infused CD8 CAR-T cells from responding patients exhibited genes associated with memory phenotype, expansion, and persistence. In contrast, genes involved in effector differentiation were responsible for a lack of clinical response. In addition to an effector signature, CAR-T cells from nonresponders displayed involvement of the PI3K–AKT–mTOR signaling pathway responsible for a more differentiated phenotype, reduced metabolic plasticity, and proliferation [277,278,279]. Collectively, the prevalence of naïve or memory T cells over effector subtypes of T cells in infused CAR-T cells has been found to be associated with adoptive T cell expansion and persistence and to predict good clinical outcome [275,280]. In accordance with these findings, Zhang et al. [276] proposed a highly personalized model for creating proper *ex vivo* stimulation to achieve the desired CAR-T cell products depending on the starting T cell phenotypes. Such an adjustment of adoptive cell therapy is certainly an innovative strategy aimed at overcoming T cell-based therapeutic challenges in CLL and holds promise for the improvement of CAR-T cell therapy in CLL (Fig. 3).

Although clinical trials in CLL showed some potential of CAR-T cell therapy in improving outcomes, its aggressive complications limited clinical expectations [281]. In fact, CAR-T cell therapy in CLL can induce several acute immune-mediated toxicities, such as cytokine release syndrome, immune effector cell-associated neurotoxicity syndrome, as well as secondary T cell lymphoma [282]. Moreover, clinical responses to CAR-T cells were lower in CLL in comparison to other indolent B cell lymphomas [283]. In CLL, T cell profound defects and inhibitory properties of the TME significantly hinder the efficacy of CAR-T cells [27]. Lisocabtagene maraleucel (liso-cel) is the first second-generation CAR-T cell product clinically evaluated in R/R CLL. The largest prospective study to date (TRANSCEND CLL004 phase I/II trial; NCT03331198) showed an ORR of 47% with 20% CR, including high-risk patients [284]. Although this regimen can induce clonal stability of the infused CAR-T cells, durable response was observed only in some patients. In turn, after a combination of CAR-T cells with ibrutinib (BTKi), the treatment outcomes were highly improved with 83% ORR, and MRD negativity was achieved in the BM of 63% of patients and in the PB of 86% of patients [284]. The combination of liso-cel with ibrutinib could be a new very promising approach in patients with heavily pretreated CLL [284]. Remarkably, due to the contradictory results of clinical trials regarding efficacy and safety profiles, the CAR-T cell regimen has not yet been approved by the EMA for the clinical management of CLL in the EU. Although in March 2024 the FDA approved liso-cel for the therapy of R/R patients with CLL progression after at least two lines of BTKi and BCL-2i therapy, this decision was accelerated and confirmatory trials are warranted to verify this preliminary approval. Nonetheless, FDA approval makes liso-cel the first CAR-T cell therapy available for CLL outside of clinical trials in the USA.

### 3.8 Bispecific T Cell Engagers

Bispecific T cell engagers (BiTEs) are a type of antibody with a capacity to bind both T cells and a tumor-specific antigen on B cells. Bispecific antibodies (BsAb) target CD3 on T cells and CD19 or CD20 on B cells, and a result of CLL-T cell interaction is malignant B cell destruction. Although several bispecific antibodies are advanced in development, thus being approved for B cell non-Hodgkin lymphoma treatment (e.g., epcoritamab, mosunetuzumab, glofitamab), none has been approved for CLL therapy so far. However, some BiTEs (e.g., blinatumomab, epcoritamab, mosunetuzumab, glofitamab, plamotamab, nebratamig) are currently being investigated in more than ten CLL trials, giving the promising results [8]. As mentioned above, blinatumomab combined with lenalidomide is currently under investigation in patients with R/R CLL (NCT02568553).

## 4 The Impact of the TME on Targeted Therapy

The CLL TME is located in the LNs, spleen, and bone marrow (BM), and plays a key role in providing pro-survival and proliferative signals to malignant B cells. In addition to its supportive role for CLL cells, TME-mediated immunosuppression—resulting from impaired anti-tumor T-cell function and upregulated Treg and MDSC subsets—facilitates the immune escape of CLL cells from surveillance, thus promoting progression and relapse of the disease. The introduction of targeted oral therapies inhibiting BTK and BCL-2 has highlighted the role of the TME in biology and the clinical outcomes of CLL. Surrounding cells in the TME, such as mesenchymal stromal cells (MSCs), nurse-like cells (NLCs), monocytes/macrophages, T cells, and NK cells are involved in facilitating the survival and proliferation of CLL cells [229]. Importantly, the CLL TME also mediates several mechanisms of drug resistance, particularly to BTK and BCL-2 inhibitors. While novel targeted therapeutics are highly effective, the TME creates ‘sanctuary’ sites (in the BM and LNs) protecting CLL cells from spontaneous and drug-mediated apoptosis and strengthening resistance. It has been demonstrated that stromal cells in these sites, specifically NLCs and MSCs, produce soluble factors (CXCL12, CXCL13, BAFF, APRIL) that facilitate CLL cell proliferation and resistance to apoptosis, thus comprising the efficacy of the targeted therapies (BTKi, BCL-2i) [229,285,286,287]. There is growing evidence that even when BTK is inhibited, signaling pathways, such as PI3K/AKT and MAPK supporting the activation of NF-kB transcription factor, can be sustained and reactivated by signals from the TME [287]. In fact, CLL cell adhesion to stromal cells triggers activation of NF-kB and AKT pathways and promotes CLL cell survival even in the presence of therapeutic agents. These interactions provide TME-derived stimuli that contribute to the activation of BCR and TLR pathways. Notably, IL-4 and TLR agonists have been identified as potent promoters of CLL cell survival and TME-mediated drug resistance [288,289]. Activation of these alternative pathways appears sufficient to sustain CLL cell survival and prompt the relapsed/refractory form of CLL.

Furthermore, direct contact and T cell-derived signaling (via CD40 or IL-4) from the TME can upregulate anti-apoptotic proteins (BCL-XL, MCL-1) which can bypass resistance to BCL-2i (venetoclax) and, to a lesser extent, BTKis [229,290]. Several *in vitro* studies have demonstrated that the interaction of stromal cells with CLL cells enhances the gene expression of anti-apoptotic proteins in CLL cells [291,292,293]. In fact, venetoclax resistance was reported to be largely attributed to MSC-mediated co-stimulation via the CD40/CD40L pathway and cytokines APRIL/BAFF, whereby inducing the expression of pro-survival proteins, such as BCL-XL, BCL-2, and MCL-1, which were shown to reduce CLL cell sensitiveness to venetoclax [290]. Further research has confirmed the role of adhesion of CLL cells to the stroma and their dynamic interactions in the induction and maintenance of resistance to targeted therapy with a special role assigned to extracellular vehicles (EVs) [294]. Notably, CLL-derived EVs have been shown to remodel the stroma in a way that induces a supportive phenotype. In turn, stroma-derived EVs can also enhance CLL cell survival and drug resistance. Accordingly, co-culture of CLL cells with stromal cells has demonstrated reduced efficacy of targeted inhibitors, indicating that higher drug concentrations are required to reveal their efficacy in the induction of apoptosis when compared to circulating CLL cells [295]. These data clearly indicate that the CLL TME provides stronger pro-survival and drug resistance signals than the PB.

## 5 Conclusion and Future Perspective

In conclusion, despite the induction of an antitumor response, CLL-derived T cells have been shown to participate in promoting CLL cell survival. Therefore, the role of non-malignant T lymphocytes in CLL is ambivalent: from supporting the leukemic clone to determining the efficacy of immunotherapy. When designing treatment strategies based on immunotherapeutics, it is important to consider the impact on the T cell population in order not to worsen the patient’s clinical outcome. Although the introduction of BTKis and BCL-2is to CLL treatment has significantly changed the treatment landscape, CLL remains an incurable disease. Currently, the most significant challenges in CLL therapy remain acquired mutations conferring drug resistance on malignant cells, the prevalence of which increases with both the duration of treatment and the number of therapeutic strategies used in individual patients. However, the observation that not all patients relapse with BTK or BCL-2 mutations highlights the importance of alternative mechanisms related to genes encoding proteins regulating apoptosis. Identification of these alternative factors could lead to the development of new targeted therapies for CLL. Their presence may significantly impair the results of current targeted therapy and prompt the development of more complex drug combinations. Furthermore, our recent data on the association between genetically determined abnormalities in the expression of anti-apoptotic p27*^KIP1^* and cyclin D2 regulating cell turnover in CLL-derived T cells and the clinical outcome indicate the need to extend observations to this non-malignant lymphocyte population. Moreover, the above studies may contribute to the development of research into targeted therapies for TME components, whose participation in the development of drug resistance is undeniable.

## Data Availability

Not applicable.

## Abbreviations

The following abbreviations are used in this manuscript:

CLL	chronic lymphocytic leukemia
BTK	Bruton’s tyrosine kinase
BCL	B-cell lymphoma
LN	lymph node
PB	peripheral blood
MDSC	myeloid-derived suppressor cell
Tfh	follicular T helper cell
Treg	regulatory T cell
TME	tumor microenvironment
CAR-T	chimeric antigen receptor
CD	cluster of differentiation
BCR	B-cell receptor
TLR	toll-like receptor
BTKi	BTK inhibitor
BCL-2i	BCL-2 inhibitor
PFS	progression-free survival
OR	overall response
CR	complete remission
OS	overall survival
MAIT	mucosa-associated immature T cell
iNK	innate natural killer
TCR	T cell receptor
Ig	immunoglobulin
MHC	major histocompatibility complex
MR1	MHC class I-related protein
CDR	complementarity-determining region
MHC	major histocompatibility complex
APC	antigen presenting cell
ZAP-70	zeta-associated protein-70
IGHV	Immunoglobulin Heavy Chain Variable region
CMV	cytomegalovirus
HLA	human leukocyte antigen
CTLA-4	cytotoxic T lymphocyte antigen-4
LAG-3	lymphocyte activation gene 3
TIGIT	T-cell immunoreceptor with Ig and ITIM domains
ITIM	immunoreceptor tyrosine-based inhibitory Motif
PD-1	programmed cell death protein 1
TIM-3	T-cell immunoglobulin mucin 3
KLRG-1	killer cell lectin-like receptor G1
PDX	patient-derived xenograft
IFN	interferon
IL	interleukin
TNF	tumor necrosis factor
Teff	effector T cell
Tem	effector memory T cell
Tcm	central memory T cell
Texh	exhausted T cell
Tmp	memory-precursor T cell
Tpex	precursor of exhausted T cell
Texh-eff	effector-like exhausted exhausted T cell
Texh-tem	terminally differentiated exhausted T cell
BLIMP	B-lymphocyte-induced maturation protein
Temra	terminally differentiated effector
TOX	thymocyte selection-associated HMG box protein
SLO	secondary lymphoid organ
CXCR	CX-chemokine receptor
CCR	CC-chemokine receptor
TBET	T-box transcription factor
GATA	guanine-adenine-thymine-adenine
ROR	retinoic acid receptor-related orphan receptor
FoxP3	Forkhead box protein 3
MAPK	mitogen-activated protein kinase
ITK	interleukin-2–inducible T-cell–associated kinase
AKT	protein kinase B
mTOR	mammalian target of rapamycin
PBMC	peripheral blood mononuclear cell
PMN	polymorphonuclear
TCL1	T-cell leukemia/lymphoma 1
LDH	lactate dehydrogenase
CDKN1B	cyclin dependent kinase inhibitor 1B
CCND2	cyclin D2
CIT	chemoimmunotherapy
TN	treatment-naive
R/R	relapsed/refractory
uMDR	undetectable Minimal Residual Disease
FDA	U.S. Food and Drug Administration
EMA	European Medicines Agency
Ven-O	venetoclax plus obinutuzumab
BiTE	bispecific T cell engager
BsAb	bispecific antibody
BM	bone marrow
MSC	mesenchymal stromal cell
NLC	nurse-like cell
BAFF	B-cell activating factor of the TNF family
APRIL	a proliferation-inducing ligand
MLC	myeloid cell leukemia
